# Effects of Different Surface Treatments of Woven Glass Fibers on Mechanical Properties of an Acrylic Denture Base Material

**DOI:** 10.3390/ijms24020909

**Published:** 2023-01-04

**Authors:** Zdravko Schauperl, Luka Ivanković, Leonard Bauer, Sanja Šolić, Marica Ivanković

**Affiliations:** 1 Faculty of Mechanical Engineering and Naval Architecture, University of Zagreb, Ivana Lučića 5, 10000 Zagreb, Croatia; 2 Faculty of Chemical Engineering and Technology, University of Zagreb, Trg Marka Marulića 19, 10001 Zagreb, Croatia; 3 Department of Mechanical Engineering, University North, J. Križanića 31b, 42000 Varaždin, Croatia

**Keywords:** dentures, DSC, FTIR, glass fibers, mechanical properties, polymer composite, polymethylmethacrylate (PMMA), treatments, acid, base, silane

## Abstract

Silanized glass fibers are popular reinforcements of acrylic denture base materials. To increase the number of surface hydroxyl groups and to improve interfacial adhesion between the matrix and reinforcements, acid or base treatments of glass fibers are commonly performed before the silanization. However, limited data are available on the effect of these treatments on the mechanical properties of acrylic matrix composite materials used for denture base applications. In this work, before the silanization of a woven glass fiber fabric (GF) with 3-(trimethoxysilyl) propyl methacrylate, activation pretreatments using HCl and NH_4_OH aqueous solutions have been performed. To characterize the glass surface, FTIR spectroscopy was used. Specimens of cured acrylic denture base resin and composites were divided into five groups: (1) cured acrylic denture base resin-control group; (2) composite with non-silanized GF; (3) composite with silanized GF; (4) composite with NH_4_OH activated and silanized GF; (5) composite with HCl activated and silanized GF. The flexural and impact properties of specimens were evaluated by means of three-point-bending tests and Charpy impact testing, respectively. The residual reactivity of the samples was analyzed using differential scanning calorimetry. The results of mechanical testing showed that acid and base pretreatments of the glass fabric had a positive effect on the flexural modulus of prepared composites but a negative effect on their impact strength. Possible interfacial adhesion mechanisms and the diffusion control of isothermal cure reactions due to vitrification have been discussed.

## 1. Introduction

Despite advances in preventive dentistry, edentulism is still a big public health problem worldwide [1]. Although there is a growing interest in dental implants, there is still a need for mobile dentures, partial or complete. Materials for dentures must meet a number of requirements since the denture in the oral cavity is exposed to chewing forces, changes in temperature, and changes in pH, and saliva is rich in enzymes and bacteria. During use, the material must remain inert, not dissolve in the oral cavity, have a neutral taste and smell, and be dimensionally stable.

The most commonly used materials in prosthetic dentistry are those based on poly (methyl methacrylate) (PMMA) owing to a reasonable cost, ease of processing and good esthetic properties [2]. However, PMMA denture base is relatively brittle and easily breaks if the patient applies high masticatory forces or if an accident happened (e.g., a denture has been dropped on a hard surface when cleaning, coughing or sneezing, in traffic accidents, sports, etc.) [2,3,4]. Frequent fractures of the denture base represent not only an inconvenience but also an additional cost to patients.

In the past decades, extensive research efforts have been made to develop novel composite materials. Polymer-matrix composites (PMC) have been reported as an excellent alternative to metals and ceramics in automotive, aerospace, building or medical applications. The range of PMC applications continues to grow and increase in diversity with every new development, as shown in recent review papers [5,6]. Fiber reinforcements are used primarily to improve strength, stiffness, fracture resistance, impact and fatigue resistance of PMC. The fiber surface conditions significantly influence the fiber-matrix interfacial bond, which in turn, determines the mechanical properties of composites. The strength properties achieved from fiber reinforcement depend also on fiber orientation [7]. To obtain optimal composite structures, more efficient manufacturing processes, higher quality products, advanced computational tools and artificial-intelligence modeling have been applied [5,8,9].

The scientists have focused their attention on fiber-reinforced PMMA composite materials to improve the denture base’s mechanical properties [10,11,12,13,14,15]. Among the available fibers (glass, aramid, carbon, nylon, ultrahigh-modulus polyethylene), glass fibers are the most popular due to their transparency and beneficial surface chemistry, which allows their adhesion to the denture base resin [15]. The adhesion of fibers is primarily based on the presence of hydroxyl groups on the surface of glass fibers and the reaction of the groups with resin monomers via silane coupling agents [16,17]. Acid or base treatments of glass substrates are commonly performed before the silanization to increase the number of surface hydroxyl groups. Increased impact [18,19,20] and fatigue strengths [21] have been reported with the use of woven glass fibers for the reinforcement of PMMA denture base materials, compared to the unidirectional fibers.

Limited data on the acid or base surface treated glass fibers effects on the mechanical properties of PMMA matrix composite materials used for denture base applications are available. This work studied the activation pretreatments of a woven glass-fiber fabric, using HCl and NH_4_OH aqueous solutions and their effects on mechanical properties of PMMA/woven glass fibers composite materials. Possible interfacial adhesion mechanisms and the diffusion control of isothermal cure reactions due to vitrification have been discussed.

## 2. Results

### 2.1. FTIR Analysis

**Figure 1:**
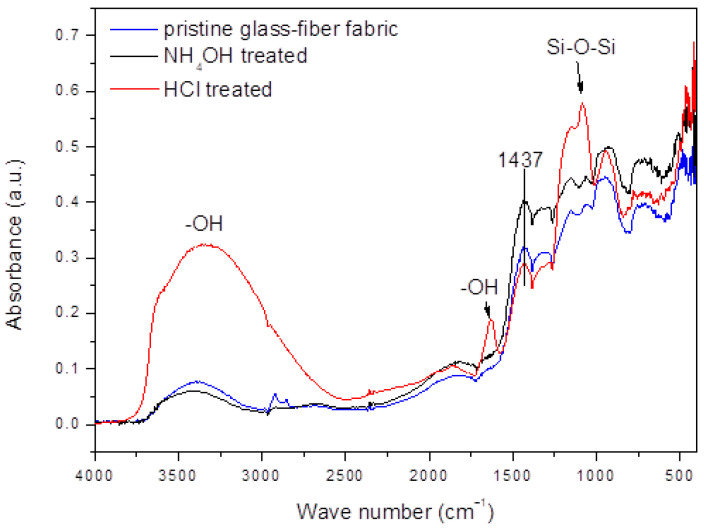
FTIR spectra of the pristine, the NH_4_OH-treated, and the HCl-treated glass-fiber fabric.

FTIR spectra of the pristine, NH_4_OH-treated, and HCl-treated woven glass-fiber fabric are compared in Figure 1.

The FTIR spectrum of NH_4_OH-treated glass-fiber fabric is very similar to the FTIR spectrum of untreated glass-fiber fabric, i.e., no changes in chemical bonds due to the base treatment are observed. Compared to the FTIR spectrum of the pristine glass-fiber fabric, the FTIR spectrum of HCl-treated glass-fiber fabric showed increased intensities of the absorption band at 1633 cm^−1^ and the broad band, with a peak at 3335 cm^−1^, related to the vibrations of OH bonds. This suggests that the glass surface is becoming increasingly hydrated and hydroxylated due to the acid treatment [22]. The increased band intensity at 1080 cm^−1^, related to the Si–O–Si siloxane bonds, and the decreased band intensity at 1437 cm^−1^ associated with other oxides (such as aluminum oxide, boron oxide, and so on, present in E glass fibers), indicate the leaching of soluble oxides from the glass fibers [23]. Such leaching may be responsible for the generation of reactive hydroxyl ions at the fiber surface, facilitating the formation of siloxane bonds.

The FTIR spectra of silanized NH_4_OH- or HCl-pretreated glass-fiber fabric were very similar to the spectra of non-silanized NH_4_OH- or HCl-treated glass-fiber fabric. This may indicate that the amount of the silane coupling agent on the glass surface is below the detection limit of the equipment.

### 2.2. Mechanical Testing

Specimens of cured acrylic denture base resin and composites were divided into five groups: (1) cured acrylic denture base resin-control group (PMMA); (2) composite with non-silanized glass fibers (PMMA/GF); (3) composite with silanized glass fibers (PMMA/GF_S); (4) composite with NH_4_OH activated and silanized glass fibers (PMMA/GF_ NH_4_OH _S); (5) composite with HCl activated and silanized glass fibers (PMMA/GF_ HCl _S). 

Flexural and impact properties of specimens were evaluated by means of three-point-bending tests and Charpy impact testing, respectively.

**Figure 2:**
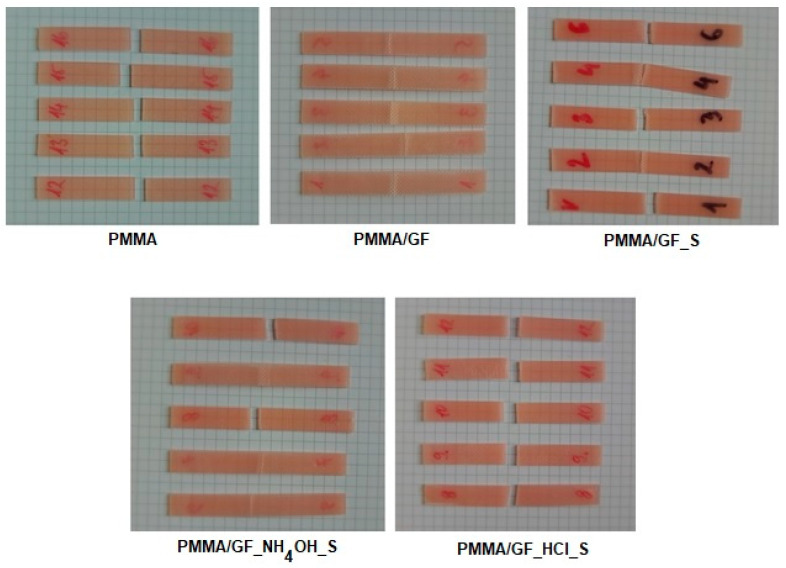
The appearance of the specimens after the three-point bending test. PMMA-cured acrylic denture base resin-control group; PMMA/GF -composite with non-silanized glass fibers; PMMA/GF_S -composite with silanized glass fibers; PMMA/GF_ NH_4_OH _S -composite with NH_4_OH activated and silanized glass fibers; PMMA/GF_ HCl _S composite with HCl activated and silanized glass fibers.

The appearance of the specimens after the three-point bending test is shown in Figure 2.

As shown in Figure 2, all the PMMA specimens had fractured into two pieces showing a rectilinear and smooth fracture surface. Visual inspection of PMMA/GF specimens indicated that only one layer of the matrix was broken, while the second matrix layer and the glass fiber layer were preserved. In the vicinity of the rupture, the broken matrix layer was partially separated from the fiber layer. Three of the five PMMA/GF_S composite specimens had fractured into two pieces, and in the other two (marked as 2 and 4), two parts of specimens are held together by some unbroken glass fibers. In two of the five specimens of the PMMA/GF__NH_4_OH_S group, there was a complete fracture of the composite, and in the other three there was a fracture of only one layer of the matrix, as seen in the PMMA/GF group. All composite specimens of PMMA_/GF_HCl had fractured into two pieces.

**Figure 3:**
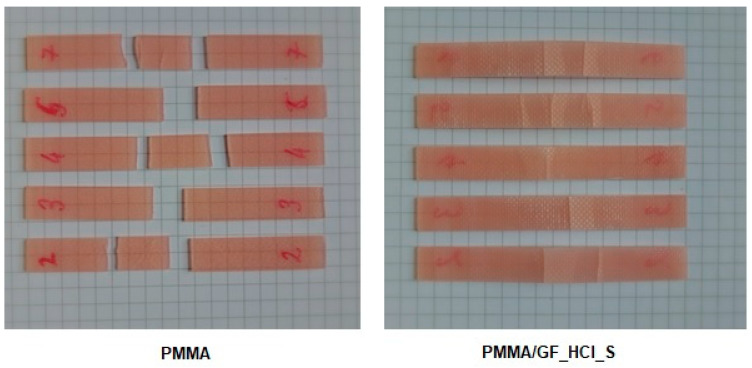
The appearance of the specimens after impact testing. PMMA-cured acrylic denture base resin-control group (specimens had fractured into two or three pieces); PMMA/GF_ HCl _S composite with HCl activated and silanized glass fibers (only one matrix layer had fractured, at one or more places, and the second matrix layer and the glass fiber layer were preserved). In the vicinity of the ruptures, the broken matrix layer was partially separated from the fiber layer.

Photographs of the two groups of specimens after the impact testing are shown in Figure 3.

During the impact testing, the PMMA specimens had fractured into two or three pieces. The PMMA/GF_HCl_S photo illustrates the composite specimens’ typical appearance after impact testing. In all composite specimens, only one matrix layer had fractured, at one or more places, while the second matrix layer and the glass fiber layer were preserved. In the vicinity of the ruptures, the broken matrix layer was partially separated from the fiber layer.

**Figure 4:**
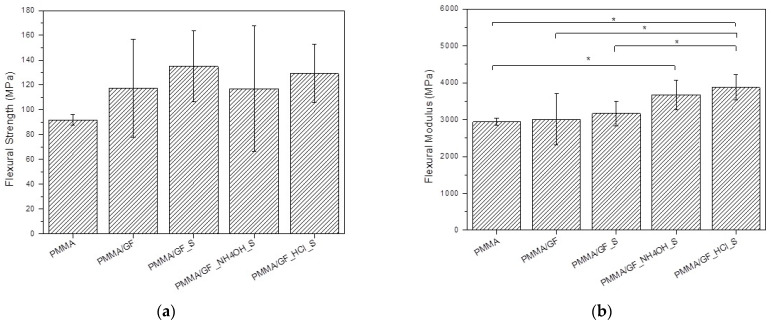

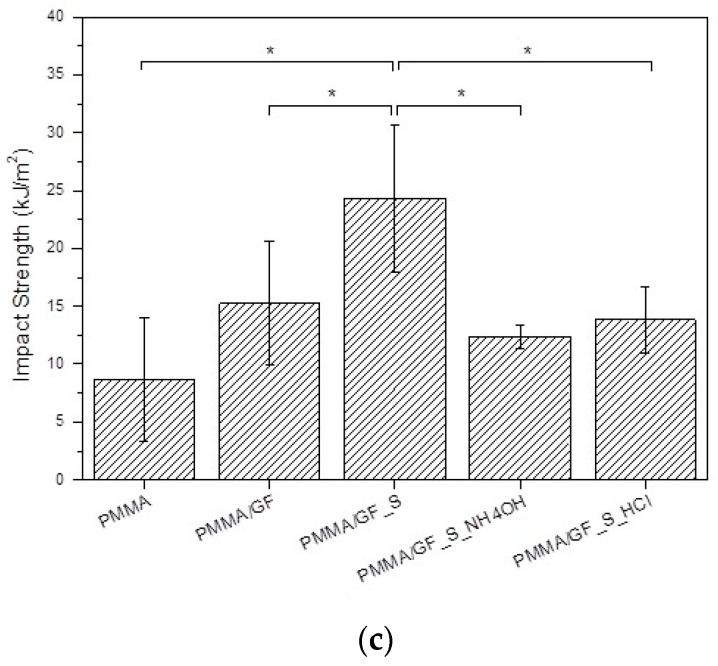
Mechanical properties of the test specimens: (**a**) Flexural strength; (**b**) Flexural modulus; (**c**) Impact strength. The significant difference between two groups is denoted by asterisk (*) with *p* < 0.05.

The flexural strength, flexural modulus, and impact strength for tested groups are compared in Figure 4a–c, respectively. The mean values and standard deviations of flexural strength, flexural modulus, and impact strength of investigated groups of specimens are presented in Table 1.

The mean flexural strength values of all groups of the reinforced specimens were higher, but not significantly, when compared to the unreinforced PMMA matrix. The highest value of flexural strength is shown in the PMMA/GF_S group, followed by the PMMA/GF_ HCl _S group.

The mean flexural modulus values of reinforced specimens PMMA/GF and PMMA/GF_S were higher, but not significantly, when compared to the unreinforced PMMA matrix. The highest values of flexural modulus are shown in the PMMA/GF_ HCl _S group (3.87 ± 0.34 GPa), which are significantly higher when compared to the unreinforced PMMA matrix, and composite specimens PMMA/GF and PMMA/GF_S, but not significantly different compared to the PMMA/GF_ NH_4_OH _S group (3.67 ± 0.40 GPa). Further, there were statistically significant differences in the flexural modulus of the control PMMA specimens and PMMA/GF_ NH_4_OH _S specimens.

The lowest mean impact strength value belonged to the control PMMA matrix, which is not significantly different compared to PMMA/GF, PMMA/GF_ NH_4_OH _S and PMMA/GF_ HCl _S. The highest mean impact strength (24.31 ± 6.34 kJ/m^2^) is shown in the PMMA/GF_S group, which is significantly higher compared to the control PMMA matrix and reinforced specimens PMMA/GF, PMMA/GF_ NH_4_OH _S and PMMA/GF_ HCl _S. A comparison between the PMMA/GF_S and PMMA/GF_ HCl _S groups shows similar values of flexural strength but superior values of flexural modulus for the PMMA/GF_ HCl _S composite. On the contrary, the acid pretreatment of glass fabric has been associated to a significant decrease in the impact strength of the PMMA/GF_ HCl _S composites, compared to PMMA/GF_S.

### 2.3. DSC Analysis

**Figure 5:**
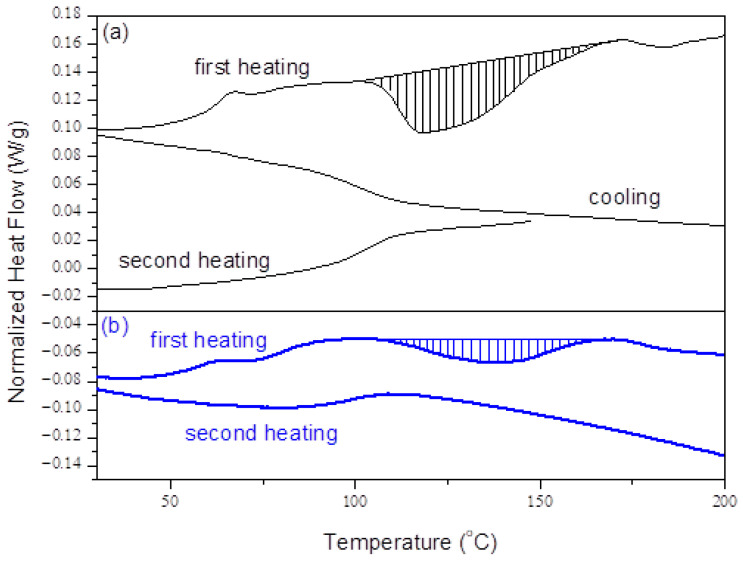
Dynamic DSC curves obtained for unreinforced acrylic systems (PMMA), previously cured at room temperature (**a**) or 55 °C (**b**) for 2 h. Heating/cooling rate: 5 °C/min.

Figure 5 shows dynamic DSC curves obtained for unreinforced acrylic systems (PMMAs), that were previously cured at room temperature (Figure 5a) or 55 °C (Figure 5b) for 2 h. The samples were heated to around 250 °C, then cooled down to room temperature and immediately reheated to 150 or 200 °C.

DSC curves obtained during the first heating show glass transition as an endothermic shift over a temperature interval between cca. 50 °C and 70 °C and an exothermic peak between 110 °C and 170 °C, indicating residual reactivity of the system. As expected, the area of the exothermic peak, corresponding to the residual heat of the reaction, is larger for the sample cured at room temperature compared to the sample cured at 55 °C. The DSC curves obtained by cooling and reheating show only the glass transition of completely cured material.

## 3. Discussion

The adhesion of glass fibers to the denture base resin is primarily based on the presence of hydroxyl groups on the surface of glass fibers, and the reaction of the groups with resin monomers via silane coupling agents. To increase the number of surface hydroxyl groups, acid or base treatments of glass substrates are commonly performed before the silanization.

**Figure 6:**
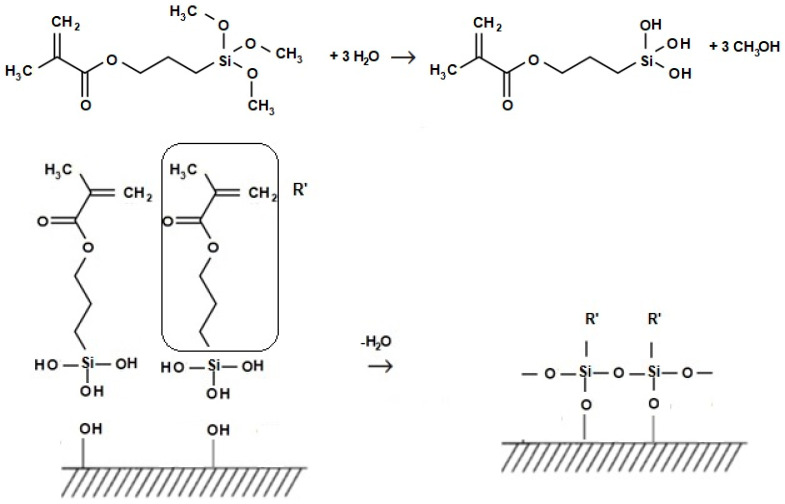
Hydrolysis of a trialkoxysilane and its reaction with a substrate with surface OH groups: vertical condensation forming covalent bonds to the substrate, and horizontal condensation forming polymeric siloxane structures.

Possible trialkoxysilane surface modification reactions are illustrated in Figure 6. During the treatment of glass fibers with the 3-(trimethoxysilyl) propyl methacrylate (3-TMSPMA), the methoxy groups can hydrolyze in an aqueous environment, producing silanol groups and liberating methanol. Silanol groups may self-condense, forming polymeric siloxane structures, and/or condense with hydroxyl groups present on the surface of glass fibers to form a covalent bond to the substrate. On the other side, the methacryloyl group of the silane coupling agent radically polymerizes with double bonds in the matrix resin and chemically binds fibers with the matrix resin.

The results of mechanical testing showed that acid and base pretreatments of the glass fiber fabric had a positive effect on the flexural modulus of prepared composites but a negative effect on their impact strength. The highest values of flexural modulus are shown in the PMMA/GF_ HCl _S group (3.87 ± 0.34 GPa), which is significantly higher when compared to unreinforced PMMA matrix (2.94 ± 0.10 GPa), and composite specimens PMMA/GF (3.01 ± 0.69 GPa) and PMMA/GF_S (3.17 ± 0.33 GPa), but not significantly different compared to the PMMA/GF_ NH_4_OH _S group (3.67 ± 0.40 GPa). Additionally, there were statistically significant differences in the flexural modulus of the control PMMA specimens and PMMA/GF_ NH_4_OH _S specimens. The highest mean impact strength (24.31 ± 6.34 kJ/m^2^) is shown in the PMMA/GF_S group, which is significantly higher compared to control PMMA matrix (8.68 ± 5.36 kJ/m^2^) and reinforced specimens PMMA/GF (15.26 ± 5.36 kJ/m^2^), PMMA/GF_ NH_4_OH _S (12.37 ± 1.04 kJ/m^2^) and PMMA/GF_ HCl _S (13.85 ± 2.87 kJ/m^2^). The impact strength of PMMA/GF_S is an order of magnitude larger than the values reported by Kanie et al. [24], and approximately twice the value reported by Dikbas et al. [25]. The flexural strength (135.1 ± 28.57 MPa) and flexural modulus (3.17 ± 0.33 GPa) of the PMMA/GF_S composite are comparable to the values reported by Kanie et al. [24].

Many factors have an impact on the mechanical properties of composites and cannot be considered in an isolated way. As seen from the FTIR results, the pretreatment of glass fibers with HCl resulted in an increase of hydroxyl groups that may facilitate chemical bonding, via silane coupling agents, and adhesion between the glass fibers and polymer matrix. Furthermore, it can be assumed that HCl etching increases the surface roughness of the fibers providing mechanical interlocking. However, the HCl pretreatment can also weaken the structure of glass fibers due to the leaching ions from the fiber surface as well as from the fiber bulk [22,23]. Our results suggest that the flexural modulus of composites depends much more on the adhesion between the glass fabric and matrix, while the microstructure of the fibers has a pronounced influence on the impact strength of composites.

Dynamic DSC characterization indicated a residual reactivity of the polymer matrix. The incomplete curing obtained in isothermal conditions can be explained in terms of diffusion control effects in the vicinity of isothermal vitrification. The structural changes produced by the polymerization reactions are associated with an increase of the glass transition temperature, Tg, of the reactive system. When the increasing Tg approaches the isothermal cure temperature, the molecular mobility is strongly reduced, and the reaction becomes diffusion controlled and eventually stops. Subsequent exposure to temperatures greater than the previous isothermal cure temperature results in the increase of the molecular mobility and further reaction. 

The presence of unreacted residual monomers in denture base acrylic resins is undesirable since they are leached out from the denture base into the saliva and transferred to the oral structures, causing adverse allergic reactions [26]. Therefore, a post curing of the denture base materials at a temperature slightly higher than the Tg of the completely cured system (cca. 110 °C) can be recommended.

Our future research will be focused on optimizing the glass fiber pretreatments, using different concentrations of HCl solutions with different time periods. The effect of post curing on the mechanical properties of the acrylic matrix composite materials will be evaluated, as well.

## 4. Materials and Methods

In this study, commercially available self-curing acrylic resin as a denture base material (Meliodent^®^ Rapid Repair, powder and liquid, Kulzer GmbH, Hanau, Germany) was used. The composition of the material, as provided by the manufacturer, is presented in Table 2.

Woven glass fibers (Satin Weave E Glass, density 163 g/m^2^, Kelteks, Karlovac, Croatia) were used to reinforce the denture base material. 3-(trimethoxysilyl) propyl methacrylate silane (98%, Acros Organics, Geel, Belgium), hydrochloric acid (35%, LACH-NER) were of reagent-grade quality and were used without further purification.

### 4.1. Surface Functionalization of Glass Fibers

A mat of woven glass fibers was cleaned by immersion in ethanol for 24 h. After drying in air, the activation pretreatment of glass fiber mat was performed using hydrochloric acid (HCl) and ammonium hydroxide (NH_4_OH) aqueous solutions, both of concentration 1 mol dm^−3^, for 24 h. Woven glass fibers were rinsed with deionized water (five consecutive immersions for 15 min each) followed by drying at room temperature for 24 h. Dried fabric was treated with a 2% (*v*/*v*) solution of 3-(trimethoxysilyl) propyl methacrylate silane (TMSPMA) in ethanol for 24 h at room temperature.

### 4.2. FTIR Analysis

Diffuse reflectance Fourier-transform infrared (DRIFT) spectroscopy was performed by Bruker Vertex 70 spectroscope. E-glass fibers were manually chopped, mixed with KBr (Sigma-Aldrich, St. Louis, MO, USA) in the mass ratio 25/75, and placed in a sample cup. Spectra were collected over the 4000 to 400 cm^−1^ range with 4 cm^−1^ resolution and 32 scans.

### 4.3. Preparation of Composite Materials

The preparation of composite materials turned out to be quite demanding because curing reactions already took place at room temperature. Due to this, the mixing of denture base materials and impregnating the fibers had to be fast, while the resin had a relatively low viscosity. Meliodent resin denture base material was hand mixed, using a powder-to liquid ratio of 10 g to 10 mL. Precut, single-layer woven glass fiber mat was placed in a glass mold between two poured layers of resin. A brush was used to incorporate the resin into the fibers and to remove air from the composite. The mold was covered with a clean glass plate and kept at room temperature for 20 min under 9.8 N load, and then at 55 °C for 2 h. Specimens of cured acrylic denture base resin, without fiber reinforcement, were also prepared as a control. Prepared materials were classified into five groups: (1) control group-cured acrylic denture base resin, without fiber reinforcement (PMMA); (2) composite with non-silanized glass fibers (PMMA/GF); (3) composite with silanized glass fibers (PMMA/GF_S); (4) composite with NH_4_OH activated and silanized glass fibers (PMMA/GF_ NH_4_OH _S); (5) composite with HCl activated and silanized glass fibers (PMMA/GF_ HCl _S). A minimum of five rectangular specimens (1.2 × 10 × 80 mm) were produced for each type of material.

### 4.4. Mechanical Testing

#### 4.4.1. Flexural Strength Test

All groups of specimens were tested for flexural strength with a three-point bending test with a universal testing machine (Inspekt Table Blue 20 kN, Hegewald and Peschke Meß- und Prüftechnik GmbH, Germany) at a crosshead speed of 5 mm/min and a span length of 20 mm. The flexural strength, *R_mf_*_,_ and the flexural modulus, *E*, were calculated with the Equations (1) and (2), respectively:

(1) $$R_{mf}=\frac{3\cdot F_{\max}\cdot L}{2\cdot b\cdot h^{2}}$$

(2) $$E=\frac{F_{1}\cdot L^{3}}{4\cdot b\cdot h^{3}\cdot d}$$

where *R_mf_* is flexural strength (MPa), *F*_max_ is the fracture load applied (N), *L* is the span length (mm), *b* is the specimen width (mm), and *h* is the specimen thickness (mm). *F*_1_ is the load at a point in the straight-line portion of the load/displacement curve [N], and *d* is the deflection at load *F*_1_ [mm].

#### 4.4.2. Charpy Impact Test

The impact strength was evaluated using the Charpy method. The impact test was performed with unnotched rectangular specimens at room temperature in an impact testing machine (Karl Frank GmbH, Weinheim-Birkenau, Germany), with a pendulum energy of 10 kpcm (approximately 1 J) at room temperature. The specimens were horizontally positioned with a distance of 62 mm between the two fixed supports. The Charpy impact strength of each test specimen was calculated using the Equation (3).

(3) $$a_{cU}=\frac{10^{3}E_{c}}{b\cdot h}$$

where *a_cU_* is Charpy impact strength of the unnotched specimen (kJ/m^2^), *E_c_* is corrected energy absorbed by breaking, or in some cases damaging the test specimen (J), *h* is thickness of specimen (mm) and *b* is width of specimen (mm).

### 4.5. DSC Analysis

The dynamic DSC analysis of cured specimens was performed on DSC 3500 Sirius^®^ Differential Scanning Calorimeter (NETZSCH-Gerätebau GmbH, Selb, Germany) at a heating/cooling rate of 5 °C/min in a nitrogen atmosphere.

### 4.6. Statistical Analysis

The data were statistically analyzed using a one-way analysis of variance (ANOVA) followed by Tukey’s post-hoc method (*p* < 0.05).

## Figures and Tables

**Table 1:** The mean values and standard deviations of flexural strength, flexural modulus, and impact strength of investigated groups of specimens. The values followed by the same letter are not significantly different from each other.

Title 1	Flexural Strength (MPa) Mean ± SD	Flexural Modulus (GPa) Mean ± SD	Impact Strength (kJ/m^2^) Mean ± SD
PMMA	91.9 ± 4.3 a	2.94 ± 0.10 a	8.68 ± 5.36 a
PMMA/GF	117.4 ± 39.5 a	3.01 ± 0.69 a	15.26 ± 5.36 a
PMMA/GF_S	135.1 ± 28.57 a	3.17 ± 0.33 a	24.31 ± 6.34 b
PMMA/GF_ NH_4_OH _S	117.0 ± 50.5 a	3.67 ± 0.40 b	12.37 ± 1.04 a
PMMA/GF_ HCl _S	129.3 ± 23.5 a	3.87 ± 0.34 b	13.85 ± 2.87 a

**Table 2:** Composition of denture base material (manufacturer information).

Material	Composition
Meliodent^®^ Rapid Repair Powder	methacrylate copolymers
Meliodent^®^ Rapid Repair Liquid	methyl methacrylate, >90%,
	Tetramethylene dimethacrylate ≥ 1–≤5%
	2-(2H-Benzotriazol-2-yl)-4-methylphenol, ≥0.25–<1%
	*N*,*N*-Dimethyl-*p*-toluidine, <1%

## Data Availability

The data presented in this study are available on request from the corresponding author.
